# Biomedical Big Data and Artificial Intelligence in Blood

**DOI:** 10.1093/gpbjnl/qzaf043

**Published:** 2025-05-02

**Authors:** Fuhong He, Zhaojun Zhang, Xiangdong Fang, Qian-Fei Wang

**Affiliations:** China National Center for Bioinformation, Beijing 100101, China; Beijing Institute of Genomics, Chinese Academy of Sciences, Beijing 100101, China; University of Chinese Academy of Sciences, Beijing 100049, China; China National Center for Bioinformation, Beijing 100101, China; Beijing Institute of Genomics, Chinese Academy of Sciences, Beijing 100101, China; University of Chinese Academy of Sciences, Beijing 100049, China; China National Center for Bioinformation, Beijing 100101, China; Beijing Institute of Genomics, Chinese Academy of Sciences, Beijing 100101, China; University of Chinese Academy of Sciences, Beijing 100049, China; China National Center for Bioinformation, Beijing 100101, China; Beijing Institute of Genomics, Chinese Academy of Sciences, Beijing 100101, China; University of Chinese Academy of Sciences, Beijing 100049, China

The hematopoietic system has long served as an excellent model for biological and medical research, owing to its highly organized hierarchical structure, accessibility for sampling, and rapid cellular turnover. These features have enabled pivotal discoveries in stem cell biology, oncogenic transformation, and targeted therapies, exemplified by milestones such as the identification of the *BCR-ABL* fusion gene and the successful development of molecular-targeted treatments. With its intrinsic advantages, recently hematology continues to provide critical insights attributed to the rapid expansion of high-throughput omics technologies and bioinformatics, and is entering a new era that emphasizes data-driven discovery and intelligent clinical decision-making.

This special issue of *Genomics, Proteomics & Bioinformatics*, entitled “Biomedical Big Data in Blood”, includes 19 studies that collectively highlight the power of how omics integration and artificial intelligence (AI) technologies are reshaping hematological research (**Table 1**). These contributions cover resource construction, mechanistic exploration, translational applications, and computational modeling. Due to space limitations, we described 10 representative studies below.

**Table 1:** Original studies, resources, and tools reported in the special issue on biomedical big data in blood

Category	Name	Brief description	Web link	Corresponding author(s)	Ref.
Resource	HemAtlas	A multi-omics hematopoiesis database	https://ngdc.cncb.ac.cn/hematlas	Feng Liu, Zhang Zhang	[2]
	EryDB	A comprehensive database for erythropoiesis and erythroid-related diseases	https://ngdc.cncb.ac.cn/EryDB/home	Xiangdong Fang, Hongzhu Qu, Yiming Bao	[3]
	NeoTCR	An immunoinformatic database for neoantigen-specific TCRs across various cancer subtypes	http://neotcrdb.bioxai.cn	Jian Liu, Kankan Wang	[4]
	HLAtyping	An HLA database to search for HLA allele frequencies across populations	http://bigdata.ibp.ac.cn/HLAtyping	Shunmin He, Tao Xu	
	HemaCisDB	A database for analyzing *cis*-regulatory elements across hematopoietic malignancies	https://hemacisdb.chinablood.com.cn	Yuxuan Liu	
	PlateletBase	A knowledgebase for platelet research and disease insights	http://plateletbase.clinlabomics.org.cn	Dongsheng Wang, Jian Huang, Yang Zhang	
	–	A review of biological data resources and machine learning applications in hematology research	https://doi.org/10.1093/gpbjnl/qzaf021	Dong Wang, Yan Huang	
Tool	HemaScope	A tool for analyzing single-cell and spatial RNA-seq data of hematopoietic cells	https://zhenyiwangthu.github.io/HemaScope_Tutorial https://ngdc.cncb.ac.cn/biocode/tool/7725 https://github.com/ZhenyiWangTHU/HemaScopeR/	Saijuan Chen, Zhu Chen, Hai Fang, Jianfeng Li	[1]
	UNISOM	A workflow for CHIP detection from WGS and WES data	https://ngdc.cncb.ac.cn/biocode/tool/7816 https://github.com/shulanmayo/UNISOM	Eric W. Klee, Shulan Tian	
	DyNDG	A dynamic network-based model, integrating DEGs to identify leukemia-related genes	https://ngdc.cncb.ac.cn/biocode/tool/BT7617 https://github.com/CSUBioGroup/DyNDG	Min Li, Ju Xiang	[9]
	HematoMap	An R-based package for identifying and visualizing leukemia lineage aberrations from single-cell and bulk RNA-seq data	https://ngdc.cncb.ac.cn/biocode/tool/7581 https://github.com/NRCTM-bioinfo/HematoMap	Saijuan Chen, Kankan Wang, Xiao-Jian Sun, Hai Fang	[10]
Original research	–	Identified G9a inhibitors as a promising therapeutic avenue for *SETD2*-mutant leukemia	https://doi.org/10.1093/gpbjnl/qzaf035	Qian-Fei Wang, Gang Huang	[5]
	–	Revealed the role of m^6^A in regulating cell state transition in normal hematopoiesis and leukemogenesis	https://doi.org/10.1093/gpbjnl/qzae049	Haojian Zhang, Ying Cheng, Fuling Zhou	[6]
	–	Found elevated levels of lactate and H3K18la, as well as their association and roles in T-ALL	https://doi.org/10.1093/gpbjnl/qzaf029	Yu Liu, Yangyang Xie, Meng Yin	[7]
	–	Revealed ITGA2B as a key regulator of heart health in high-altitude settlers	https://doi.org/10.1093/gpbjnl/qzaf030	Yue Gao, Wei Zhou, Haitao Lu	[8]
	–	Identified B-cell clone types and established a clonal threshold specific for each patient clonality profile	https://doi.org/10.1093/gpbjnl/qzaf041	María José Terol, Ana-Bárbara García-García	
	–	Demonstrated the specificity of genetic regulation of platelet traits in the context of β thalassemia	https://doi.org/10.1093/gpbjnl/qzae065	Xiangmin Xu, Zilin Li	
	–	Developed a non-invasive liquid biopsy assay for early-stage diagnosis of breast cancer with cfDNA fragmentomics	https://doi.org/10.1093/gpbjnl/qzaf028	Jianzhong Su, Zhihua Liu, Xiang Wang	
	–	Discovered clonal hematopoietic mutations in plasma cell disorders	https://doi.org/10.1093/gpbjnl/qzaf027	Jian Li, Chunyan Sun	

*Note*: HLA, human leukocyte antigen; DEG, differentially expressed gene; CHIP, clonal hematopoiesis of indeterminate potential; RNA-seq, RNA sequencing; TCR, T-cell receptor; T-ALL, T-cell acute lymphoblastic leukemia; cfDNA, cell-free DNA; WGS, whole-genome sequencing; WES, whole-exome sequencing; AI, artificial intelligence.

## Data integration and bioinformatic tools: building the digital foundation

Robust data resources and user-friendly analytical platforms are essential for advancing omics-driven hematology research. This section features four articles that focus on the development of specialized tools and databases addressing diverse cellular contexts, model organisms, and clinical scenarios.

Wang et al. [1] developed HemaScope, an open-source bioinformatics toolkit tailored for analyzing single-cell and spatial transcriptomic data in hematopoietic systems. HemaScope integrates modules for atlas construction, lineage tracing, dynamic transcriptional analysis, and microenvironmental profiling. The toolkit has demonstrated robust performance across multiple scenarios, including bone marrow aging, acute myeloid leukemia (AML), and T-cell lymphoma. Kang et al. [2] introduced HemAtlas, a cross-species, multi-omics database dedicated to hematopoiesis. It incorporates transcriptomic, epigenomic, and spatial transcriptomic data from humans, mice, zebrafish, and *in vitro* hematopoietic stem and progenitor cell models. HemAtlas enables comparative analyses across developmental stages and tissues, enhancing understanding of hematopoietic development and regeneration.

Zheng et al. [3] established EryDB, a comprehensive erythroid transcriptomic database integrating bulk and single-cell RNA-seq datasets across species and disease conditions. EryDB facilitates comparative exploration of erythropoiesis and identification of dysregulated pathways in erythroid-related disorders. Zhou et al. [4] developed NeoTCR, an immunoinformatic database of experimentally validated neoantigen-specific T-cell receptors (TCRs) from 18 cancer types. NeoTCR offers unified annotations of publicly available neoantigen-specific TCR sequences along with relevant neoantigen information. It also provides a one-stop platform for clonotype discovery, neoantigen annotation, and immunotherapy prediction, bridging sequencing data with precision immuno-oncology.

Collectively, these resources help close existing gaps in data accessibility, analytical capabilities, and clinical integration, laying a digital foundation for mechanistic discoveries and personalized therapeutic strategies.

## Omics-guided mechanistic studies: from molecular insights to therapeutic strategies

The convergence of multi-omics technologies and clinical expertise is transforming our understanding of hematologic diseases, bridging molecular mechanisms with therapeutic advances. As mechanistic research enters the big data era, validation and translational foresight become crucial. This section highlights four studies that leverage genomics, transcriptomics, epigenomics, and integrative omics to uncover disease pathways and therapeutic strategies.

Epigenetic reprogramming is recognized as one of the major drivers in hematological malignancies. Zhang et al. [5] identified histone methyltransferase G9a inhibitors as candidate drugs for SETD2-deficient leukemia using the Connectivity Map combined with a drug screening platform. Their integrative analysis of transcriptomic and ChIP-seq data showed that G9a inhibition upregulates *let-7a-2*, potentially via H3K9me2 reduction, suppressing oncogenic MYC signaling. This epigenetic regulation–noncoding RNA–oncogenic axis positions G9a as a promising therapeutic target. Liu et al. [6] revealed that RNA *N*^6^-methyladenosine (m^6^A) modification affects hematopoietic stem cell differentiation and leukemogenesis. They found that ABCD2, as an m^6^A-regulated driver of AML progression, promotes leukemogenesis by modulating fatty acid metabolism and maintaining leukemia cell viability. These findings highlight the central role of RNA epigenetics in stem cell fate and malignant transformation, paving the way for novel epitranscriptomic therapeutic strategies. Wu et al. [7] reported that lactate generated by the Warburg effect induces histone H3K18 lactylation in T-cell acute lymphoblastic leukemia (T-ALL), marking super-enhancer regions and activating oncogenes such as *IGFBP2*. This metabolism–epigenetics–transcription axis redefines the oncogenic role of lactate through chromatin remodeling, and suggests that targeting lactate-driven H3K18 lactylation could represent a promising therapeutic avenue for T-ALL.

Interestingly, one study exemplifies the synergy between traditional Chinese medicine and modern omics technologies in generating actionable translational insights. Wang et al. [8] applied plasma proteomics to investigate high-altitude-induced myocardial injury and identified ITGA2B as a regulator of *IL-6* expression and metabolic reprogramming under hypoxia. Tanshinone IIa, a compound derived from traditional Chinese medicine, reversed ITGA2B-mediated myocardial injury.

Together, these studies underscore the power of integrative omics with detailed clinical phenotyping in decoding hematologic pathogenesis and guiding precision medicine. As the field evolves, sustained collaboration between omics researchers and clinicians remains essential to translate these discoveries into meaningful benefits for patients.

## AI empowering: advancing hematological research and clinical translation

The integration of AI into hematological research has enabled advances in both biological understanding and clinical decision-making. This special issue highlights cutting-edge applications of AI models that decode disease complexity and bridge computational predictions with clinical utility. Two contributions featured here present innovative AI-driven strategies for identifying regulatory drivers and refining disease classification.

A et al. [9] developed the DyNDG model, which integrates machine learning and dynamic network modeling. By constructing a time-series multilayer network and applying a random-walk-based propagation framework, DyNDG captures temporal changes in gene interactions and identifies leukemia-related genes with higher accuracy. This strategy enhances the discovery of stage-specific biomarkers and emphasizes the value of temporal dynamics in gene prioritization. Dai et al. [10] presented HematoMap, a platform combining AI and single-cell omics to quantify lineage aberrancy and infer leukemic origins. Using cosine similarity and LASSO regression, HematoMap maps transcriptional deviations from bulk RNA-seq data. This enables personalized risk stratification and links molecular subtypes to clinical outcomes, demonstrating the broad applicability of AI in translational hematology.

In summary, these studies exemplify the dual impact of AI: resolving biological complexity through advanced pattern recognition and promoting clinical innovation through predictive modeling.

## Outlook: a new era of mechanistic discoveries and intelligent decision-making

This special issue reflects rapid progress in hematology, driven by multi-omics data resources and integration as well as computational tools. Future progress will rely on three pillars: standardized data curation, interpretable intelligent algorithms, and deep investigation of biological mechanisms. To promote openness and reproducibility, we strongly encourage contributors to share their datasets and analytical tools through national infrastructure platforms like the National Genomics Data Center (NGDC) of China. Such efforts will promote data interoperability and foster collaborative innovation across the hematology research community.

## CRediT author statement

**Fuhong He:** Conceptualization, Writing – original draft, Writing – review & editing. **Zhaojun Zhang:** Conceptualization, Writing – original draft, Writing – review & editing. **Xiangdong Fang:** Conceptualization, Writing – review & editing. **Qian-Fei Wang:** Conceptualization, Writing – review & editing. All authors have read and approved the final manuscript.
